# Dentate gyrus neurons that are born at the peak of development, but not before or after, die in adulthood

**DOI:** 10.1002/brb3.1435

**Published:** 2019-10-01

**Authors:** Tina Ciric, Shaina P. Cahill, Jason S. Snyder

**Affiliations:** ^1^ Department of Psychology Djavad Mowafaghian Centre for Brain Health University of British Columbia Vancouver BC Canada

**Keywords:** cell death, development, neurogenesis, ontogeny, plasticity, turnover

## Abstract

**Introduction:**

In the dentate gyrus of the rodent hippocampus, neurogenesis begins prenatally and continues to the end of life. Adult‐born neurons often die in the first few weeks after mitosis, but those that survive to 1 month persist indefinitely. In contrast, neurons born at the peak of development are initially stable but can die later in adulthood. Physiological and pathological changes in the hippocampus may therefore result from both the addition of new neurons and the loss of older neurons. The extent of neuronal loss remains unclear since no studies have examined whether neurons born at other stages of development also undergo delayed cell death.

**Methods:**

We used BrdU to label dentate granule cells that were born in male rats on embryonic day 19 (E19; before the developmental peak), postnatal day 6 (P6; peak), and P21 (after the peak). We quantified BrdU^+^ neurons in separate groups of rats at 2 and 6 months post‐BrdU injection to estimate cell death in young adulthood.

**Results:**

Consistent with previous work, there was a 15% loss of P6‐born neurons between 2 and 6 months of age. In contrast, E19‐ or P21‐born neurons were stable throughout young adulthood.

**Discussion:**

Delayed death of P6‐born neurons suggests these cells may play a unique role in hippocampal plasticity adulthood, for example, by contributing to the turnover of hippocampal memory. Their loss may also play a role in disorders that are characterized by hippocampal atrophy.

## Graphical abstract

In contrast to dentate gyrus neurons born in adulthood, neurons born in development die once animals have reached adulthood. Here, we find that only neurons born at the peak of development undergo delayed cell death in adulthood.

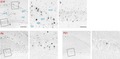

## 1 INTRODUCTION

Proposed functions of dentate gyrus neurogenesis have been inspired by patterns of cell addition and loss. In particular, neuronal survival has been repeatedly linked to behavior and cognitive demand. At baseline, a large proportion of adult‐born neurons die within the first few weeks after mitosis (Cameron, Woolley, McEwen, & Gould, 1993; Kempermann, Gast, Kronenberg, Yamaguchi, & Gage, 2003). Learning and environmental stimulation can rescue adult‐born neurons at specific stages of cellular development, typically around 1–2 weeks of cell age (Anderson, Sisti, Curlik, & Shors, 2010; Epp, Spritzer, & Galea, 2007; Gould, Beylin, Tanapat, Reeves, & Shors, 1999; Tashiro, Makino, & Gage, 2007). Learning also causes death of specific cohorts of immature neurons, suggesting that both the addition and removal of neurons are involved in the adaptive response to experience (Dupret et al., 2007; Olariu, Cleaver, Shore, Brewer, & Cameron, 2005). Survival of immature DG neurons is competitive and depends on NMDA receptors (Tashiro, Sandler, Toni, Zhao, & Gage, 2006). Since mature adult‐born neurons selectively respond to stimuli that they encountered at immaturity (Kee, Teixeira, Wang, & Frankland, 2007), they may store information as long as they survive, which appears to be indefinite (Dayer, Ford, Cleaver, Yassaee, & Cameron, 2003; Kempermann et al., 2003).

In contrast to our detailed understanding of the dynamics of neurogenesis in adulthood, much less is known about neurons born in early development, even though this is when the majority of DG neurons are born (Snyder, 2019). Specifically, while there have been many studies of DG development, few studies have examined the properties of age‐defined cohorts of developmentally born DG neurons. We recently examined the short‐ and long‐term survival of rat DG neurons born at the peak of development, P6 (Cahill, Yu, Green, Todorova, & Snyder, 2017). Whereas adult‐born neurons have an early critical period for survival, P6‐born neurons did not. Also, while adult‐born neurons are stable after reaching 4 weeks of age, we found that 17% of P6‐born neurons died between 2 and 6 months of age. The functional relevance of this delayed cell death is unknown, but it could contribute to forgetting and hippocampal/DG atrophy in disorders such as depression (Boldrini et al., 2013; Mahar et al., 2017; McKinnon, Yucel, Nazarov, & MacQueen, 2009). Whether delayed cell death is observed among cells born at ages other than P6 is unknown.

Mature DG neurons are not identical and even cells born at different stages of perinatal development can have distinct properties (Kerloch, Clavreul, Goron, Abrous, & Pacary, 2018; Save, Baude, & Cossart, 2018; Snyder, 2019). Indeed, DG neurogenesis spans many developmental milestones that may differentially recruit new neurons and affect their survival phenotype (e.g., birth, eye opening, weaning). To determine the scope of delayed cell death, we therefore examined long‐term survival of DG neurons born at three different stages of rat development: (a) DG granule neurons born on embryonic day 19. These are largely generated by proliferative cells that are migrating from the primary to secondary germinal zones, outside of the dentate gyrus (Altman & Bayer, 1990a). They are among the earliest granule neurons added to the DG. (b) DG granule neurons born on postnatal day 6. These cells are born in the tertiary germinal zone, which is located in the dentate hilus (Altman & Bayer, 1990b). Postnatal day 6 is within the peak of DG development, so cells born at this time represent a large proportion of DG neurons (Schlessinger, Cowan, & Gottlieb, 1975). This population is known to undergo cell death in young adulthood (Cahill et al., 2017; Dayer et al., 2003). (c) DG granule neurons born at P21. These neurons are generated in the subgranular zone, at the border between the granule cell layer and the hilus (Altman & Bayer, 1990b). Adult neurogenesis also occurs in the subgranular zone. However, rats at P21 are sexually immature, still acquiring hippocampal learning and memory abilities (Akers & Hamilton, 2007; Raineki et al., 2010) and are therefore fundamentally distinct from adults.

## 2 METHODS

Except where stated otherwise, methodological details are identical to those of our recent study of cell death in P6‐born cells (Cahill et al., 2017). To label cells born prenatally (E19 group), rats were generated from timed pregnancies. Male and female breeders were paired each afternoon, and females were inspected for the presence of sperm the following morning. The day when sperm was detected was designated as E1, and at this time, females were separated from the males and housed singly for the remainder of the pregnancy. On E19, mothers were injected once with the thymidine analog BrdU (50 mg/kg, subcutaneous; Roche) to label proliferative cells that are beginning to migrate to the DG. Mothers remained with the litters for 3 weeks after birth, when offspring were weaned to 2 per cage. For the P6 and P21 groups, breeder pairs remained together from conception to age P21, when offspring were weaned 2/cage. For postnatal groups, the day of birth was designated as postnatal day 1. Pups were injected with BrdU on postnatal day 6 (50 mg/kg, intraperitoneal) or postnatal day 21 (200 mg/kg, intraperitoneal). Rats in the P21 group were injected as they were weaned into their new cages. Groups of rats from each condition (E19, P6, P21) were perfused at 8 weeks and 6 months post‐BrdU injection. Thus, cell ages were identical across studies but animal ages varied slightly. We refer to the 8‐week group as “2 months” for simplicity. Hippocampi were processed for BrdU immunohistochemistry as previously described (Cahill et al., 2017).

Total BrdU^+^ cell counts were estimated from coded slides with the optical fractionator technique and stereological counting principles (West, Slomianka, & Gundersen, 1991), using an Olympus BX53 light microscope equipped with a motorized stage and software for stereological sampling (StereoInvestigator; MicroBrightField). Dentate gyrus granule cell layer areas were outlined at 2× magnification, and cell counting was performed at 40× magnification. The section sampling frequency for all groups was 1/12. Other sampling parameters were optimized to accommodate differences in cell density across groups. Specifically, for the E19 group the sampling grid was 380 × 380 μm, the counting frame was 70 × 70 μm, and the dissector height was 13 μm with a 3‐μm guard zone. For the P6 group, the sampling grid was 220 × 220 μm, the counting frame was 70 × 70 μm, and the dissector height was 8‐μm with a 4‐μm guard zone. For the P21 group, the sampling grid was 100 × 100 μm, the counting frame was 60 × 60 μm, and the dissector height was 13 μm with a 3‐μm guard zone.

## 3 RESULTS

**Figure 1:**
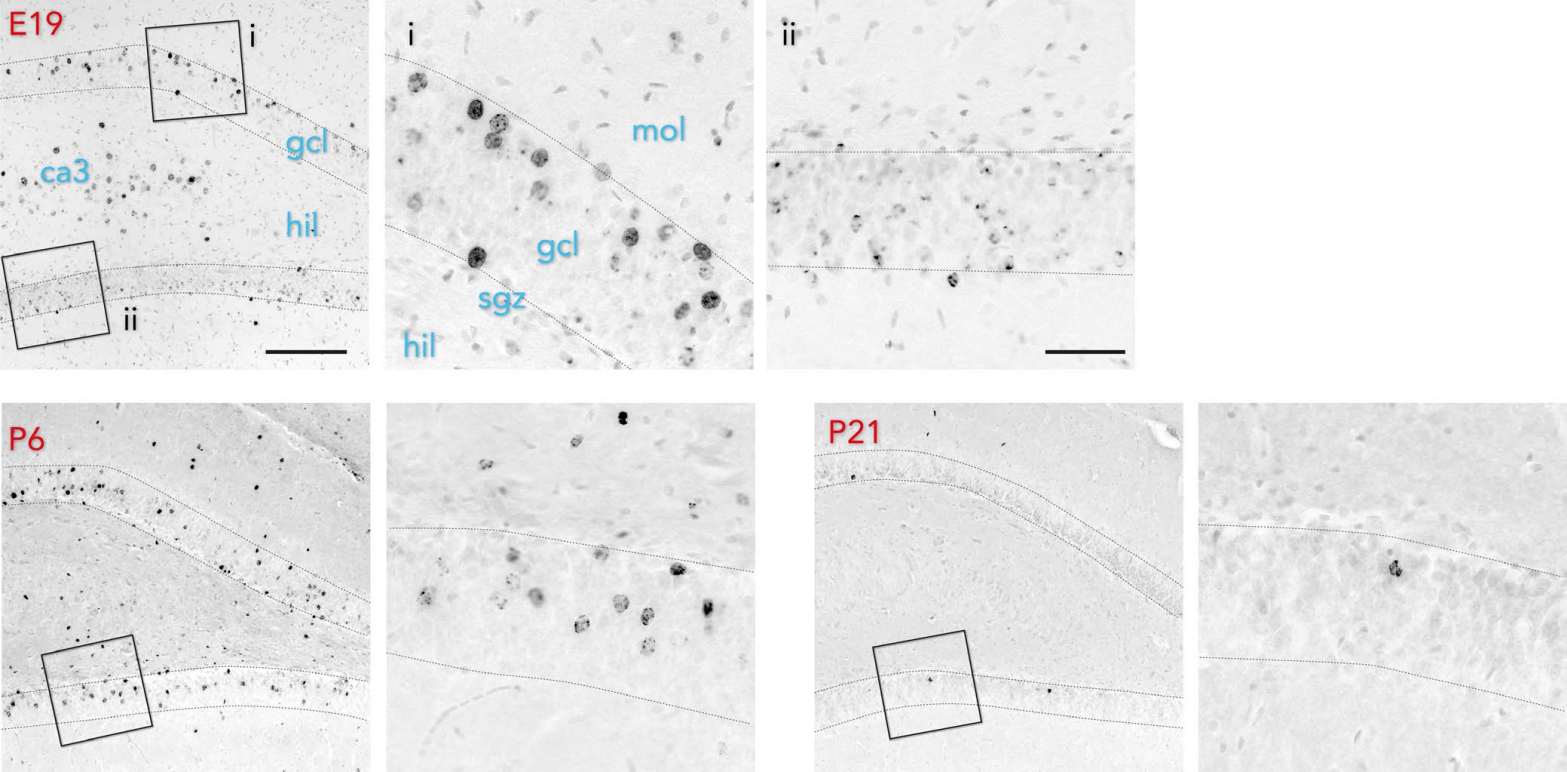
BrdU labeling of developmentally born cells. E19 injections labeled granule neurons in the superficial granule cell layer, near the molecular layer. Strongly labeled cells can be seen in inset i. Weakly labeled, speckled cells can be seen in inset ii. P6 injections labeled cells primarily in the middle of the granule cell layer. P21 injections labeled many fewer cells that were located in the deep portion of the granule cell layer, near the hilus/subgranular zone. gcl, granule cell layer; hil, hilus; mol, molecular layer; sgz subgranular zone. Scale bars 200 μm

BrdU injections resulted in age‐related patterns of cell labeling that were consistent with previous studies of rodent development (Figure 1). Injections at E19 labeled CA3 and CA1 pyramidal neurons and DG neurons in the superficial granule cell layer of the dorsal DG. Granule neurons were clearly identifiable by their large round nuclei and dense packing in the granule cell layer. In the ventral DG, BrdU^+^ cells were located in deeper portions of the granule cell layer consistent with generation of granule neurons beginning at ~E14 and proceeding along ventral to dorsal, and superficial to deep, axes (Altman & Bayer, 1990a; Schlessinger et al., 1975). E19‐born neurons were either darkly or lightly stained for BrdU. Darkly labeled cells most likely reflect cells that were undergoing mitosis at the time of BrdU injection and lightly labeled cells are either, (a) those that were generated by subsequent divisions and underwent label dilution, or (b) those that took up BrdU for only a portion of S‐phase. P6 BrdU injections did not label any pyramidal neurons in the CA fields, as expected. In the DG, BrdU^+^ granule cells were primarily located in the middle ~50% of the granule cell layer, but labeled cells could be observed in the more superficial and deep aspects as well. P6‐born cells had less variation in BrdU staining intensity than E19 cells and most cells tended to be strongly and evenly stained throughout the nucleus. Finally, BrdU injections on P21 labeled a smaller population of cells that were primarily located in the deep 1/3 of the granule cell layer, with some cells located between them and the hilus due to continued addition of younger cohorts of granule cells after P21.

**Figure 2:**
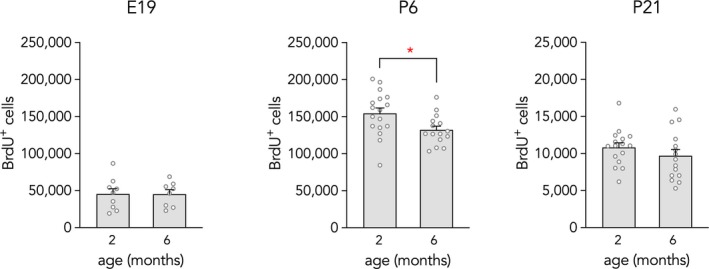
Long‐term neuronal survival. A similar number of E19‐born cells were present at 2 and 6 months of age (*T*_15_ = 0.008, *p* = .99). There was a 15% loss of P6‐born cells between 2 and 6 months of age (*T*_30_ = 2.5, *p = .02). A similar number of P21‐born cells were present at 2 and 6 months of age (*T*_29_ = 1.1, *p* = .3). Note the 10× smaller *y*‐axis scale for the P21 data. Bars represent mean ± *SE*

In rats that received BrdU injections on E19, there were darkly and lightly labeled cells. We therefore set staining intensity criteria to avoid counting cells that were labeled due to repeated division of stem cells after E19. First, we only counted cells that had ≥10% of their nucleus stained for BrdU. This revealed 163,188 cells at 2 months and 153,929 cells at 6 months (coefficient of error = 0.06; *T*_15_ = 0.5, *p* = .6). Since these numbers were comparable to our previous counts of P6‐born cells (Cahill et al., 2017), and previous reports have indicated that prenatal neurogenesis is substantially lower than early postnatal neurogenesis (Bayer & Altman, 1974; Schlessinger et al., 1975), this criterion likely included many cells that were labeled by redivision and overestimated the true value. We therefore adjusted our criterion to include only cells that were unambiguously, strongly labeled with BrdU (≥50% of the nucleus stained). Stereological quantification of intensely labeled cells revealed ~45,000 granule neurons, which fits with previous evidence that ~3× fewer cells are generated on E19 than on P6 (Schlessinger et al., 1975). Thus, this is likely a more accurate estimate of the true E19 value. While the coefficient of error increased when we only included the intensely labeled cells (0.11), since fewer cells were sampled, counts were virtually identical at the 2‐ and 6‐month time points. These results collectively indicate that prenatally born cells do not undergo delayed cell death in young adulthood (Figure 2). We found that ~150,000 granule neurons were born on P6, and there was a 15% loss of BrdU^+^ cells between 2 and 6 months of age, replicating our previous findings (Cahill et al., 2017). By P21, ~10,000 cells were labeled, which was over 10× lower than P6 levels. There was no loss of P21‐born cells from 2 and 6 months of age. Error coefficients were low for both the P6 (0.06) and P21 (0.07) groups.

## 4 DISCUSSION

These results replicate previous findings that P6‐born granule neurons undergo delayed cell death in young adulthood and they reveal that this cellular attrition is specific to neurons born around the peak of DG development. While our additional timepoints identify E19 and P21 as boundaries, the precise age window during which these “vulnerable” cells are generated, and therefore the total number that is lost, remains unclear. To our knowledge, only two studies have quantified rat DG neurogenesis throughout the early developmental period (Bayer & Altman, 1974; Schlessinger et al., 1975). Using ^3^H‐Thy, both studies found that ~15% of granule cells are added before birth and that neurogenesis is highest during the first postnatal week, with ~50% of the total population added from P1 to P8. If similar patterns of cell loss (~15%) are observed for all cells born in this window, 7.5% of the granule cell population would be lost in young adulthood, or ~180,000 cells bilaterally (assuming 2.4 million neurons in total [West et al., 1991]). Of course, fewer cells may be lost if the window is shorter, and more cells may be lost if the window is longer, than the first postnatal week. Further work is therefore needed to identify the magnitude of cell loss in order to estimate whether it may have a meaningful impact. Since these calculations apply a relative measure of change (the % loss we identified) to previous datasets, they are not confounded by possible differences between labeling methods. In contrast, it is somewhat more difficult to compare absolute cell counts between neurons born at different developmental stages, since the kinetics of BrdU labeling in early development is poorly understood. We chose 50 mg/kg BrdU for E19 and P6 since this dose is most‐commonly used in developmental studies and because direct comparisons have shown that all developmentally born ^3^H‐Thy^+^ cells are also BrdU^+^ (Miller & Nowakowski, 1988), suggesting this dose may be saturating. Similarly, we chose 200 mg/kg for P21 rats since this dose is likely to label all dividing cells in adults (Cameron & McKay, 2001). Notably, these rates of cell loss are broadly comparable to, but slightly lower, than rates of cell addition over the same timeframe, due to adult neurogenesis (345,000 cells added between 2 and 6 months [Snyder & Cameron, 2012]). Thus, continued neurogenesis may lead to overall growth in the DG (Bayer, 1982; Bayer, Yackel, & Puri, 1982), but loss of earlier‐born cells could explain why some have failed to observe age‐related increases in DG granule cell number (Boss, Peterson, & Cowan, 1985). Whether there is a causal balance between loss of developmentally born cells and addition of adult‐born cells remains to be determined.

An outstanding question is why P6‐born cells undergo delayed cell death but not cells born earlier or later in development. Assuming that there is a degree of functional heterogeneity within the DG that depends on when a cell was born (Snyder, 2019), P6‐born cells may be reflective of the largest cohort of DG neurons and therefore may be the most dispensable. Their loss could also be due to extrinsic features; for example, the type of information that they store during their critical period may predispose them to turn over. E19‐born cells, in contrast, could reflect specialized dentate gyrus cell populations (Kerloch et al., 2018; Save et al., 2018) that are more resistant to cell death. It is also likely that E19‐generated neurons are very different from the majority of DG neurons since they undergo significant migration to reach their final destination. Finally, a recent report indicates that P21‐born neurons in mice are structurally identical to adult‐born neurons (Kerloch et al., 2018), suggesting that, by weaning, neurogenesis may produce a consistent granule cell phenotype.

Ultimately, functional studies of age‐defined cohorts of neurons will be needed to determine the mechanisms and consequences of granule cell loss. The cells examined in this study are clearly developing in very different environments (which could also influence later susceptibility to cell death). E19‐born cells may be those cells that are developing afferent synapses during the 2nd postnatal week, before eye opening (Ye, Song Liu, Pasternak, & Trommer, 2000). P6‐born cells display peak immediate‐early gene expression at P21, when animals are leaving the nest (Cahill et al., 2017). Finally, P21‐born cells will likely be in their critical period of development and plasticity during adolescence and early adulthood. Early‐life experiences have lasting effects on hippocampal function (Nguyen, Bagot, Diorio, Wong, & Meaney, 2015). Thus, the retention and removal of cells that were developing during early‐life experiences may shape adaptive behavioral responses in adulthood.

## Data Availability

The data that support the findings of this study are available from the corresponding author upon request.
